# O029. Lessons from the expert patients. Advice for the physician to improve the care of cluster headache patients

**DOI:** 10.1186/1129-2377-16-S1-A94

**Published:** 2015-09-28

**Authors:** Paolo Rossi, Elena Ruiz De La Torre, Cristina Tassorelli, the European Headache Alliance

**Affiliations:** INI, Grottaferrata, Italy; European Headache Alliance, Italy; Headache Science Centre, National Neurological Institute C. Mondino Foundation, Pavia, Italy

## Background and aim

In rare disorders Expert Patients (EPs) may be used to educate physicians representing an authoritative source of information about patient needs and expectations. The aim of this study was to collect a list of recommendations from EPs for physicians engaged in the management of cluster headache (CH) with the purpose to improve their ability in taking care of CH patients.

## Method

Patients’ association providing aid and support to CH sufferers in six European countries received a letter of invitation to join the study in April 2014. Those CH groups who accepted to participate were requested to provide a list of recommendations and advice, from at least 5 EPs, for physicians engaged in the management of CH.

## Results

Eighty-three advices from 25 EPs (SP, I, UK, NL, SWE) were available for a qualitative content analysis. Seventy-seven percent of the EPs’ advices could be grouped in 7 main recommendations: 1) CH diagnosis is easy if you consider few clinical clues; 2) prescribe sumatriptan and oxygen; 3) suggest patient to avoid hiding and to be active in a patients’ support group; 4) take patient seriously and listen to him/her; 5) provide good information and be able to correct the misleading ones; 6) be sensitive to CH impact on the patient's significant one; 7) acknowledge that CH is a serious medical disorder that can have a significant impact on the person and support him/her.

## Conclusions

EPs’ recommendations should be used to propose pragmatic patient-centered changes in healthcare services dedicated to CH.

Written informed consent to publication was obtained from the patient(s).

